# Let Him That Is without Sin Cast the First Stone

**Published:** 1896-07

**Authors:** 


"LET HIM THAT IS WITHOUT SIN CAST THE FIRST STONE."—It is with surprise and regret that we notice in the June number of the *Texas Medical News* our long-time friend, Dr. J. W. McLaughlin, one of the editors, apparently loses his temper. He says some very ugly and spiteful things of us, a part of it, too,—oh my!—in bad English. To be sure, they are not true; but when defeated in argument some men get rattled, and become reckless. The resort to personalities and abuse is not the methods usually employed by gentlemen; they are coarse and vulgar, and should put one beyond the pale of recognition as a legitimate controvertist. When our esteemed contemporary applies to us such epithets as "fool or enemy to the Association," "sore-head," "chronic kicker," etc., he forgets himself, and we decline to meet him on that level.

Boasting of having "demolished" our argument, and of having "shown the fallacy" of our position, the doctor should be happy; yet, notwithstanding that triumph (?), he seems very much piqued. It is unpleasant, we know, to have the inconsistencies of one's public professional record shown up and clinched by indubitable proofs, but it does not warrant the resort to personalities and epithets. We submit there is nothing in what we have written about the doctor during the discussion of the quarantine question and the mixed board that is personal in the sense of applying to his private professional life, and certainly nothing that was intended to be offensive; hence the doctor's flying off in this unbecoming manner is a matter of both surprise and deep regret, and is unjustifiable. A man's public professional record and his published utterances, i. e., as officer, member or committee man of an association are legitimate matter for editorial criticism, and we certainly have confined ourself to these. We have made a logical argument, and in every instance have presented facts in support of it, and our readers are interested in a discussion of matters so deeply affecting their profession; but to single out some one act of indiscretion in the private professional life of an opponent, or some error of judgment, and hold it up for ridicule before ones' readers in default of argument or reply, is a species of meanness of which we had not thought our distinguished and dignified friend and contemporary capable. We could, as the doctor well knows, *retaliate in kind*, and make it a little embarrassing; but there is something about the *role* of informer that is despicable, and we will not. To fight the devil with fire is not always good policy.

The Doctor should have obeyed the Scriptural injunction at the head of this article. He is not without sin,—who of us is? —and yet he has hurled this ugly projectile,—harmless to us, it is true, but which, like the bushman's brutal weapon in unskillful hands, will surely rebound upon his own head. Those who reside in vitreous structures should be careful in the handling of missiles.

What is referred to above is the Doctor's request for Dr. Daniel to "define the ethics of a physician serving on a pension board with an irregular." * (*Texas Medical News*, June, 1896.)

We answer with pleasure: pleased to be considered authority on ethics. For a physician to serve on a pension board with an irregular,—or any other board—in his professional capacity, is decidedly unethical: from Dr. McLaughlin's standpoint *at present*, it is ethical; from his former record (see "1877 vs. 1879" herewith) it is decidedly and flagrantly unethical; sufficiently so to make a fuss about; ethical or permissible from the State Medical Association's *present* point of view; violently and unpardonably unethical and beneath the dignity of a physician, accoding to its position in 1877, and to the present time, when "expediency" seems to have outweighed all ethical considerations. Opinions differ. See letter from Dr. Davis herewith.

The unkindest thing our old friend and long-time sympathiser, in the matter referred to, has said, though, is that "the Association concluded it could dispense with Dr. Daniel's services as secretary," and "the Association failed to show appreciation of his services as secretary." Dr. McLaughlin must have been very much rattled and unguarded to make such statement. The doctor knows that the Association did not conclude it could dispense with Dr. Daniel's services, and he knows that the Association did not fail to show appreciation of his services, and he must be aware that every old member knows that such state-

[* The incident so ungenerously referred to by Dr. McLaughlin is this: Dr. Daniel was refused position on pension board because he had been a Confederate surgeon and a Confederate private. He determined to secure it or know why he was disfranchised. He succeeded. Shortly afterward a homeopath was appointed—completing the number. They met and organized and examined one man. Dr. Daniel resigned without having served, or served further, if you prefer, and without having received any compensation. It was an error into which we were unwittingly led; freely confessed and regretted. The Doctor can make the most of it—we hereby assist him, by giving his unkind insinuations the benefit of this journal's large circulation.]

ment is not in accord with the facts. It is a matter of record. The doctor knows that Dr. Daniel resigned in direct opposition to the wishes of the majority. Through the JOURNAL he had offended certain favorites by showing up certain acts of quackery, and they were clamoring for his scalp. He resigned in tne interest of harmony and pacification, an act characterized as "magnanimous," and as testifying his devotion to the Association's progress and advancement; and he was immediately reelected, and resigned a second time a year later. Just why the doctor should make the above statement, therefore—one calculated to convey a false impression, and do me an injury—is inexplicable; it must be ascribed to pique or injudicious haste and want of proper care. I feel well assured the doctor, who is, and for so long a time, has been my friend, would not intentionally do me an injury or injustice.

It is unfortunate that the discussion of subjects, in which neither of us has any personal interest, can not be carried on without acrimony. The doctor is new to the editorial harness, or he would be able to give and take in a more fraternal spirit. We drop the subject of quarantine transfer. Further discussion would be bootless, as it can not be decided which of us is right, till the test is made. We would rather the doctor would "transfer" all the quarantines in Christendom than to feel that we had wounded his *amour propre* or alienated his kindly regard.

